# Palmitoleic acid n7 improves glucose uptake and metabolism and attenuates oxidative stress in human visceral adipocytes

**DOI:** 10.1007/s12020-026-04766-6

**Published:** 2026-09-28

**Authors:** Nayara Ariel da Silva Lisboa, Nina Bruna de Souza Mawandji, Júlia Martins Vieira, Anna Clara da Silva Kefner, Pietra Morellato Oliveira, Karolini Zuqui Nunes, Paulo Henrique Oliveira de Souza, Fabiano Kenji Haraguchi, Blanca Elena Guerrero Daboin, Maria Isabel Alonso-Vale, Andressa Bolsoni-Lopes

**Affiliations:** 1 Graduate Program in Nutrition and Health, Federal University of Espírito Santo, Vitória, ES Brazil; 2 Graduate Program in Physiological Sciences, Federal University of Espírito Santo, Vitória, ES Brazil; 3 Cassiano Antônio de Moraes University Hospital, Federal University of Espírito Santo, Vitória, ES Brazil; 4 Graduate Program in Chemical Biology, Federal University of São Paulo, São Paulo, SP Brazil

**Keywords:** Obesity, Prediabetic state, Monounsaturated fatty acid, Visceral adipose tissue, Glucose metabolism, Oxidative stress

## Abstract

**Purpose:**

Obesity-associated dysfunction of visceral white adipose tissue (vWAT) is characterized by impaired glucose handling, altered adipokine secretion, and redox imbalance, contributing to metabolic deterioration. Palmitoleic acid (16:1n-7), a monounsaturated fatty acid, has been implicated in regulation of glucose and lipid metabolism in rodent and cellular models; however, its effects on human adipocytes under metabolically compromised conditions remain incompletely understood. Here, we investigated the effects of in vitro treatment with 16:1n-7 on glucose uptake and metabolism, adipokine secretion, and oxidative stress in isolated adipocytes and vWAT obtained from women with obesity and prediabetes.

**Methods:**

vWAT explants were treated in vitro with palmitoleic acid (16:1n-7) or palmitic acid (16:0) at 200 µM for 48 h. Glucose and lipid metabolism, adipokine secretion, and oxidative stress were evaluated.

**Results:**

16:1n-7 increased basal and insulin-stimulated glucose uptake in association with upregulation of GLUT1 and GLUT4 expression and increased AMPKα protein content. In parallel, 16:1n-7 promoted coordinated changes in metabolic gene expression favoring glucose utilization and glyceroneogenesis rather than de novo lipogenesis, without changes in lipolytic activity, accompanied by increased citrate synthase and PPARG expression. Endocrine function was also modulated, as 16:1n-7 reduced resistin secretion without impairing adiponectin levels. Moreover, although lipid peroxidation remained unchanged, 16:1n-7 reduced protein oxidation and reactive oxygen species production, together with increased IDH2 and reduced NOS2 expression, supporting selective attenuation of oxidative stress.

**Conclusion:**

Palmitoleic acid enhances glucose uptake through coordinated regulation of GLUT1 and GLUT4 and contributes to improved metabolic function in human adipocytes and redox homeostasis in vWAT obtained from women with obesity and prediabetes. These findings identify 16:1n-7 as a bioactive lipid that modulates human adipose tissue function under metabolically compromised conditions, underscoring its role in the nutritional regulation of glucose metabolism and redox homeostasis.

## Introduction

Obesity is associated with profound metabolic abnormalities in visceral white adipose tissue (vWAT), including impaired glucose and lipid metabolism, dysregulated adipokine secretion, and disrupted cellular redox balance, which collectively contribute to systemic metabolic deterioration and increase the risk of insulin resistance, prediabetes, and type 2 diabetes [1–5].

Despite insulin-stimulated glucose uptake predominating in skeletal muscle, healthy WAT is a meaningful contributor to systemic glucose homeostasis, accounting for approximately 10–15% of total glucose disposal [6, 7]. In white adipocytes, glucose uptake occurs through basal and insulin-sensitive transporters, primarily GLUT1 and GLUT4, respectively [8], and glucose serves as an important metabolic substrate supporting energy production, lipid synthesis and storage, and the generation of regulatory metabolites that influence redox homeostasis, membrane fluidity, and insulin sensitivity [9, 10]. However, chronic nutrient excess and inflammatory signaling in obesity reprogram these tightly coordinated processes of adipocytes, modifying substrate utilization patterns and altering intracellular signaling networks, promoting a pro-oxidative and pro-inflammatory microenvironment within vWAT, perpetuating tissue dysfunction and amplifying systemic metabolic disturbance [5, 9–12].

Given the pivotal role of vWAT in energy regulation, identifying molecules capable of restoring adipocyte metabolic and redox balance represents a promising strategy for mitigating obesity and its associated diseases. In this context, fatty acids have emerged as key signaling mediators of metabolic and inflammatory pathways. Among these, palmitoleic acid (16:1n-7), a monounsaturated omega-7 fatty acid, has been described as a lipokine due to its beneficial effects on lipid and glucose metabolism, insulin sensitivity, and inflammatory profiles across multiple target tissues, including WAT, liver, skeletal muscle, and pancreas [13–17].

Previous work from our group has shown that 16:1n-7 enhances glucose uptake and GLUT4 expression in association with AMPK activation in murine adipocytes and 3T3-L1 cells [13], increases lipolysis in a PPARα-dependent manner [14], and improves the metabolic and oxidative capacity of WAT, partially preventing obesity-induced metabolic, hypertrophic, and inflammatory alterations [18, 19]. Despite these consistent findings in experimental models, whether similar effects can be reproduced in human adipocytes under metabolically compromised conditions remains largely unexplored.

Therefore, we tested the hypothesis that palmitoleic acid positively modulates glucose metabolism and cellular redox status in human vWAT. To this end, we evaluated the effects of in vitro treatment with 16:1n-7 on glucose uptake, the expression of proteins and genes involved in glucose and lipid metabolism, adipokine secretion, and oxidative stress using isolated adipocytes and vWAT explants obtained from women with obesity and prediabetes.

## Materials and methods

### Human subjects and sample collection

vWAT samples were obtained from the omental region of women aged 25–40 years with a body mass index (BMI) of 40–45 kg/m² and clinically diagnosed prediabetes, who underwent bariatric surgery at the Hospital Universitário Cassiano Antônio Moraes (HUCAM), Vitória, ES, Brazil. The diagnosis of prediabetes was based on the American Diabetes Association (ADA) criteria and established by any of the following alterations: a fasting plasma glucose between 100 and 125 mg/dL, an HbA1c between 5.7 and 6.4%, or a 2 h plasma glucose concentration of 140–199 mg/dL during a 75-g oral glucose tolerance test [20, 21]. Exclusion criteria included prior bariatric surgery, anti-obesity medication use, diabetes, hypertension, severe cardiopulmonary disease, immunological, oncological or infectious diseases and smoking [22]. The clinical and metabolic characteristics of the donors are summarized in Table 1.

**Table 1:** Clinical and metabolic characteristics of the adipose tissue donors

Variable	Value (n = 5)
Age (Years)	35 ± 3.4
BMI (kg/m²)	44 ± 2.5
HbA1c (%)	6.0 ± 0.13
FPG (mg/dL)	99 ± 10.4
SBP (mmHg)	117 ± 6.7
DBP (mmHg)	80 ± 6.3

Data are presented as mean ± standard deviation (SD) (n = 5)

*BMI* body mass index, *FPG* fasting plasma glucose, *HbA1c* glycated hemoglobin, *SBP* systolic blood pressure, *DBP* diastolic blood pressure

Immediately after excision, vWAT samples were placed in sterile phosphate-buffered saline (PBS), maintained at 4 °C in an insulated container, and transported to the Laboratory of Cardiovascular and Adipose Tissue Studies at the Federal University of Espírito Santo (UFES), where they were processed within 30 min of collection.

All participants provided written informed consent prior to enrollment. The study protocol was approved by the Research Ethics Committee of HUCAM/UFES (No. 6.745.447; approval date: April 2024; CAAE: 59075722.7.0000.5071).

### Adipose tissue processing and fatty acids treatments

vWAT explants were cultured in 12-well plates in DMEM/F-12 containing 10% fetal bovine serum (FBS), penicillin (100 U/mL), and streptomycin (100 µg/mL) at 37 °C and 5% CO₂, in the presence of palmitoleic acid (16:1n-7, 200 µM, P9417 Sigma-Aldrich) or palmitic acid (16:0, 200 µM, P0500, Sigma-Aldrich), or vehicle (0.05% ethanol), for 48 h. Cell viability was not affected, as assessed by MTT assay (see Supplementary Figure S1). After 44 h, samples were washed with PBS and serum-starved in low-glucose DMEM without FBS, maintaining treatments, for 4 h.

Following treatment, experiments were conducted using either intact vWAT explants or isolated adipocytes in suspension obtained from the treated tissue, as appropriate for each assay. Briefly, for adipocyte isolation, vWAT explants were digested in DMEM supplemented with HEPES (20 mM), sodium pyruvate (2 mM), bovine serum albumin (BSA, 4%), and collagenase type II (1.0 mg/mL; Sigma-Aldrich), pH 7.4, at 37 °C for 40 min in an orbital shaking water bath (130 rpm) [13, 23]. Isolated adipocytes were filtered and washed three times in the same buffer without collagenase. A subset of adipocytes was photographed using an optical microscope (×100 magnification) equipped with a digital camera (Moticam 1000; Motic). Mean adipocyte diameter was determined by measuring 50 cells using Motic-Images Plus 2.0 software. These morphological measurements were subsequently used to calculate mean cellular volume (assuming a spherical shape) and estimate adipocyte number, allowing glucose uptake and lipolysis to be expressed relative to adipocyte number, as previously described [13, 14].

Five independent donors (n = 5) were included as biological replicates. For each donor, three technical replicates per treatment condition were performed, and average values per donor were used for statistical analysis.

### Basal and insulin-stimulated glucose uptake assay

Glucose uptake was assessed in isolated adipocytes using a nonradioactive assay (Glucose Uptake-Glo™ Assay, Promega; J1342), according to the manufacturer’s instructions with minor modifications. Briefly, adipocytes (10⁶ cells) were washed with PBS and incubated with 2-deoxyglucose buffer (1 mM) in the absence or presence of insulin (10 nM), for 3 min. The reaction was halted by the addition of Stop Buffer, followed by Neutralization Buffer, and samples were subsequently incubated with the 2-deoxyglucose-6-phosphate (2DG6P) Detection Reagent for 1 h. Luminescence was recorded using a Synergy™ 2 microplate reader (BioTek, Winooski, VT, USA).

### Lipolysis

Lipolysis was assessed by glycerol release into the incubation medium. Isolated adipocytes (10⁶ cells/mL) were incubated in Krebs–Ringer phosphate buffer (pH 7.4) containing BSA (20 mM) and glucose (5 mM) for 30 min at 37 °C, in the presence or absence of isoproterenol (2 × 10⁻⁶ M). Glycerol levels were measured using a commercial kit (Free Glycerol Determination Kit, Sigma).

### Adipokine dectection

Adiponectin and resistin levels were quantified in adipocyte-conditioned medium using human immunoenzymatic assay kits (ELISA, Sigma-Aldrich; RAB0005 and RAB0419), according to the manufacturer’s instructions.

### RNA extraction and quantitative real-time polymerase

Total RNA was extracted from isolated adipocytes using TRIzol reagent (Invitrogen). RNA quality was assessed by 260/280 and 260/230 ratios using a NanoDrop spectrophotometer (Thermo Scientific). cDNA was synthesized using the High-Capacity cDNA Reverse Transcription Kit with RNase Inhibitor (Thermo Scientific). Gene expression was analyzed by quantitative real-time PCR (qPCR) using a QuantStudio™3 system (Thermo Scientific) and SYBR Green dye. Relative expression was calculated using the 2 − ∆∆CT method [13] and normalized to beta-actin (*ACTB*). Primer sequences are listed in Table 2.

**Table 2:** Forward (sense) and reverse (antisense) sequences of the primers used in qPCR

Gene	Foward (5’−3’)	Reverse (3’−5’)
*ACTB*	GAGACCGCGTCCGCC	ATCATCCATGGTGAGCTGGC
*PNPLA2*	TGTCCTTCACCATCCGCTTG	GCATCACCAGGTACTGGCA
*CS*	TCCGACCCTTACCTGTCCTT	GCCAGACAAGCACTTCCTGA
*FASN*	GCAAGCTGAAGGACCTGTCT	AATCTGGGTTGATGCCTCCG
*GK*	CTCTCATAGCGTGAAAGCTGG	CCCACCAATGCAGCAGACT
*GPD1*	CGGGTGACCATGTGGGTATT	CTGGGACAGCCACCACATTT
*GLUT1*	TGGCATCAACGCTGTCTTCT	AGCCAATGGTGGCATACACA
*GLUT4*	CACCTTGGTCTCGGTGTTGT	ACGTAGCTCATGGCTGGAAC
*LIPE*	CCAGGGAGTGAAGCTAGGC	TGAGCCTTGAGGCTGTATCC
*IDH2*	TGCCTGGAGTTTAAAAGGCGA	ATAGATTTCTTATCACCCCAGTTCC
*NOS2*	TCCCGAGTCAGAGTCACCAT	TCCATGCAGACAACCTTGGG
*LPIN1*	TGGTCTTCAAACATTAACGTGC	GAGCTCCTTCACGGTGACAA
*PCK1*	ATCCCCAAAACAGGCCTCAG	ACGTACATGGTGCGACCTTT
*PPARG*	AGTCAGCCTTTAACGAAATGACC	CACGGAGCTGATCCCAAAGT

Caption: Beta-actin (*ACTB*); Adipose triglyceride lipase - ATGL (*PNPLA2*); Citrate synthase (*CS*); Fatty acid synthase (*FASN*); Glycerol kinase (*GK*); Glycerol-3-phosphate dehydrogenase 1 (*GPD1*); Glucose transporter type 1 (*GLUT1*); Glucose transporter type 4 (*GLUT4*); Hormone-sensitive lipase HSL (*LIPE*) Isocitrate dehydrogenase 2 (*IDH2*); Inducible nitric oxide synthase – *iNOS (NOS2)*; Lipin 1 (*LPIN*1); Phosphoenolpyruvate carboxykinase 1-cytosolic (*PCK1*); Peroxisome proliferator–activated receptor gamma (*PPARG)*. Source: Primer-BLAST (NCBI, 2024)

### Immunohistochemistry (IHC)

Protein expression in vWAT was evaluated by IHC as previously described [24]. vWAT samples were fixed in 4% paraformaldehyde, embedded in paraffin, and sectioned (5 μm). Sections were deparaffinized, rehydrated, and subjected to antigen retrieval in citrate buffer (pH 6.0) using microwave heating. Endogenous peroxidase activity and nonspecific binding were blocked with 3% hydrogen peroxide and 5% BSA in TBS-T, respectively. Sections were incubated overnight at 4 °C with primary antibodies against AKT, phospho-AKT (Ser473), AMPKα, and phospho-AMPKα (Thr172) (Cell Signaling Technology; #9272, #4058 L, #2532S, and #2535S), followed by an HRP-conjugated secondary antibody. Immunoreactivity was detected using DAB (Sigma-Aldrich). Sections were analyzed using a light microscope (Zeiss Axiolab 5), and immunostaining was quantified using ImageJ by measuring the raw integrated density within regions of interest (ROIs) of identical size. Data were expressed as arbitrary units (a.u.).

### Thiobarbituric acid–reactive substances (TBARS/MDA) determination

Lipid peroxidation in vWAT was assessed by TBARS, expressed as malondialdehyde (MDA), using a colorimetric method [24]. Samples were homogenized, incubated with thiobarbituric acid (1%) and phosphoric acid (7%) at 95 °C for 15 min, followed by butanol extraction and centrifugation (2000 rpm, 4 °C, 5 min). Absorbance was measured at 540 nm using a spectrophotometer (Thermo Scientific Multiskan FC). Protein content was determined by the Bradford method, and results were expressed as µmol MDA/mg of protein.

### Advanced oxidation protein products (AOPP)

AOPP levels in vWAT were assessed by measuring protein carbonyl groups [25]. Samples were homogenized in PBS and incubated with potassium iodide (1.16 M) and acetic acid, and absorbance was measured at 340 nm. Chloramine T (267 µM) was used for standard curve generation. Protein content was determined by the Bradford method, and results were expressed as µM/mg of protein.

### In situ production of reactive oxygen species (ROS)

In situ ROS production was assessed as previously described [26]. vWAT fragments were incubated with dihydroethidium (DHE, 2 µM) and DAPI (2 µM) for 30 min at 37 °C in the dark. Tiron (1 mM) was used as a negative control. Tissues were then fixed in 4% paraformaldehyde, embedded in OCT, and sectioned (20 µm). Images were acquired using a fluorescence microscope (Leica DM2500) with appropriate filter settings (DHE: Ex 535 nm/Em 610 nm). Identical acquisition parameters were used for all groups. Fluorescence intensity was quantified using ImageJ.

### Statistical analysis

Data are expressed as mean ± SEM. One-Way ANOVA followed by Tukey post-hoc test was used to compare the effects of different treatments. Analysis was performed using GraphPad Prism 8.0 software (GraphPad Software). The level of significance was set at *P* < 0.05.

## Results

### Clinical characteristics of the donors

The clinical and metabolic characteristics of the vWAT donors are summarized in Table 1. All donors were women with severe obesity and prediabetes who met the predefined eligibility criteria for inclusion in the study.

### Palmitoleic acid increases basal and insulin-stimulated glucose uptake and the expression of *GLUT1* and *GLUT4* in human adipocytes

**Fig. 1:**
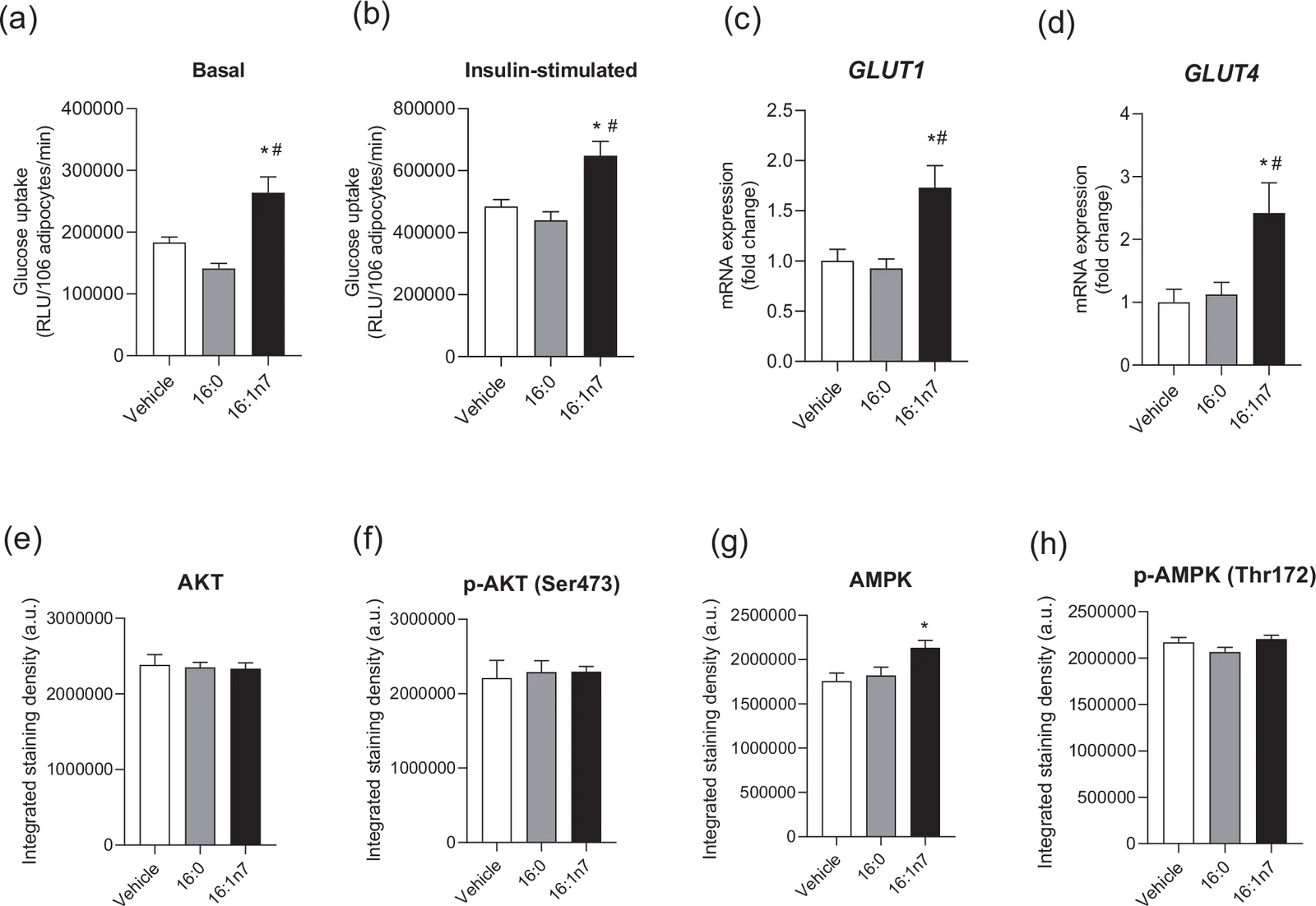
Basal and insulin-stimulated glucose uptake (**a**–**b**) and mRNA expression of glucose transporter 1 *(GLUT1*, (**c**) and glucose transporter 4 (*GLUT4*, **d**) in isolated human omental adipocytes. Immunohistochemical analysis of AKT (**e**), phospho-AKT (Ser473) (**f**), AMPKα (**g**), and phospho-AMPKα (Thr172) (**h**) in human visceral adipose tissue (vWAT) explants. Samples were treated for 48 h with either vehicle, palmitic acid (16:0, 200 μM), or palmitoleic acid (16:1n-7, 200 μM). Results are expressed as means ± SEM ($n=4{-}5$/grou*p*). *p  <  0.05 vs. vehicle and ^#^*p*  <  0^.^05 vs. 16:0

Our investigation of the effects of 16:1n-7 on human adipocyte glucose metabolism began with the assessment of glucose uptake. As shown in Fig. 1a-b, in vitro treatment with 16:1n-7, but not 16:0, induced a significant increase in glucose uptake under basal conditions by 43.9% and 86.8% compared with vehicle and 16:0, respectively. Under insulin-stimulated conditions, glucose uptake was increased by 33.9 and 47.5% relative to vehicle and 16:0, respectively. Importantly, this increase in glucose uptake induced by 16:1n-7 was associated with a marked upregulation of *GLUT1* and *GLUT4* gene expression (Fig. 1c-d).

To further elucidate the molecular pathways potentially mediating the effects of 16:1n-7 on adipocytes, we subsequently assessed the activation status of key intracellular signaling proteins involved in glucose uptake and metabolism, including AKT and AMPKα. As illustrated in Fig. 1e-h, no changes were observed in AKT protein content or p-AKT (Ser473) among the treatments; however, palmitoleic acid increased AMPKα protein content without altering p-AMPKα (Thr172).

These findings support the potential of palmitoleic acid to improve glucose uptake and *GLUT1/GLUT4* expression in human adipocytes under metabolic stress, through basal and insulin-stimulated processes without engaging canonical signaling pathways.

### Palmitoleic acid differentially regulates metabolic gene expression toward glucose utilization in human adipocytes

We next investigated whether the increase in glucose uptake induced by 16:1n-7 was accompanied by changes in the expression of genes involved in glucose and lipid metabolism, providing insight into intracellular glucose utilization.

**Fig. 2:**
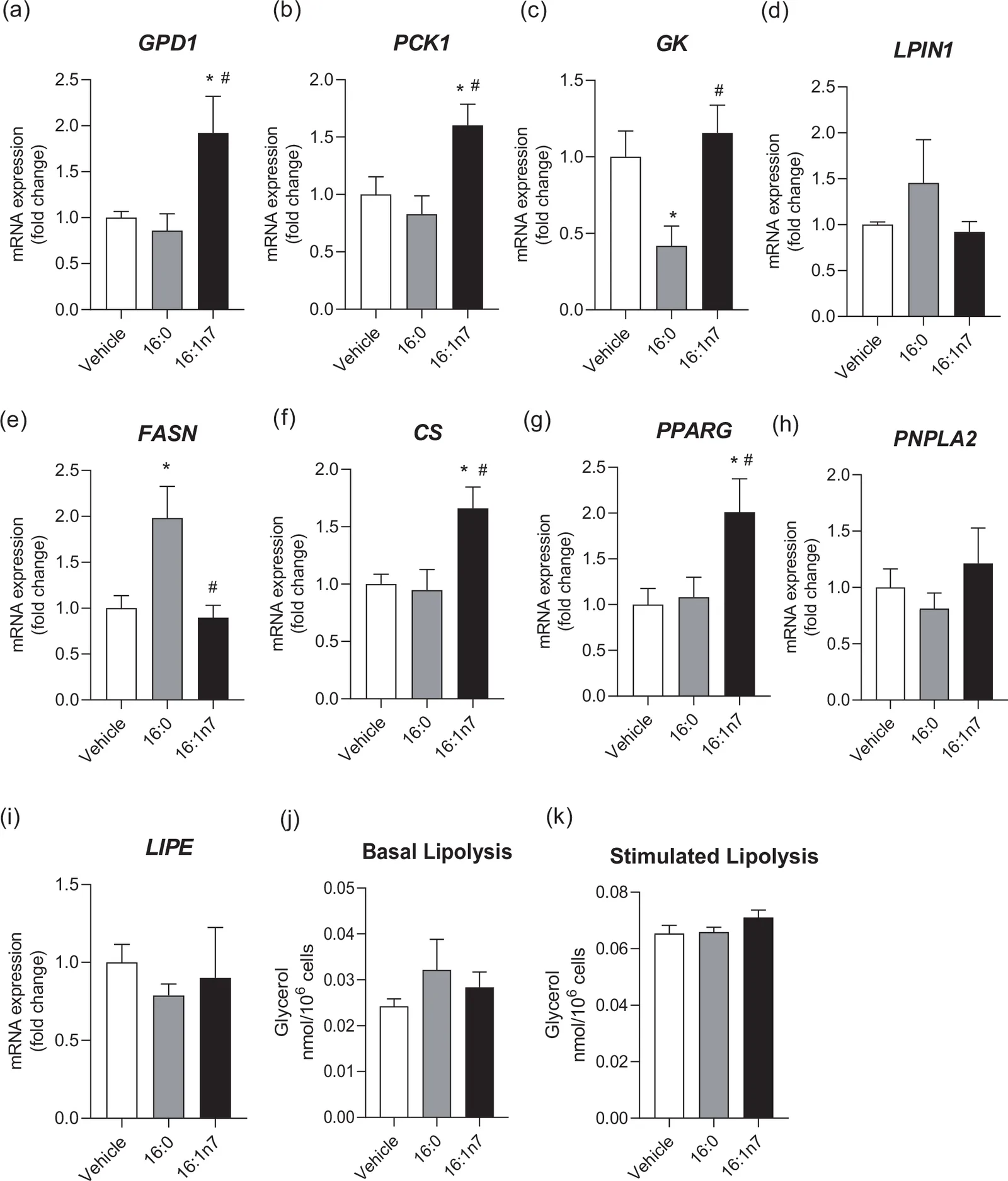
mRNA expression of Glycerol-3-phosphate dehydrogenase 1 (*GPD1*, **a**); Phosphoenolpyruvate carboxykinase 1-cytosolic (*PCK1*, **b**); Glycerol kinase (*GK*, **c**); Lipin 1 (*LPIN1*, **d**); Fatty acid synthase (*FASN*, **e**); Citrate synthase (*CS*, **f**); Peroxisome proliferator–activated receptor gamma (*PPARG*, **g**); Adipose triglyceride lipase - ATGL (*PNPLA2*, **h**); Hormone-sensitive lipase - HSL (*LIPE*, **i**); and lipolysis, assessed by glycerol release under basal (**j**) and isoproterenol-stimulated conditions (Panel k) in isolated human omental adipocytes treated for 48 h with either vehicle, palmitic acid (16:0, 200 μM) or palmitoleic acid (16:1n7, 200 μM). Results are means  ±  SEM (*n*  =  5/group). *p  <  0.05 vs. vehicle and ^#^*p*  <  0.05 vs^.^ 16:0

As shown in Fig. 2a-c, treatment with 16:1n-7 significantly increased the mRNA levels of *GPD1* and *PCK1* compared with both vehicle and 16:0, indicating a coordinated increase in glyceroneogenesis-related pathways and redirection of glucose toward glycerol-3-phosphate synthesis. In addition, *GK* expression was reduced in 16:0-treated adipocytes, whereas 16:1n-7 maintained *GK* expression at levels comparable to vehicle.

No statistically significant differences were observed in *LPIN1* expression among the treatments (Fig. 2d). However, 16:0, but not 16:1n-7, increased the expression of *FASN*, a key enzyme of de novo lipogenesis (Fig. 2e). Notably, 16:1n-7 significantly increased *CS* expression (Fig. 2f) and *PPARG* expression, whereas 16:0 had no effect (Fig. 2g).

Lipolysis, as well as *PNPLA2* and *LIPE* mRNA levels, were not altered by 16:1n-7 under the conditions tested (Fig. 2h–k). Likewise, neither palmitic acid nor palmitoleic acid affected mean adipocyte diameter after 48 h of treatment (vehicle: 104 ± 4.6 µm; 16:0: 103 ± 3.7 µm; 16:1n-7: 102 ± 3.8 µm, *P* > *0.05*).

### Palmitoleic acid alters adipokine secretion associated with glucose metabolism

**Fig. 3:**
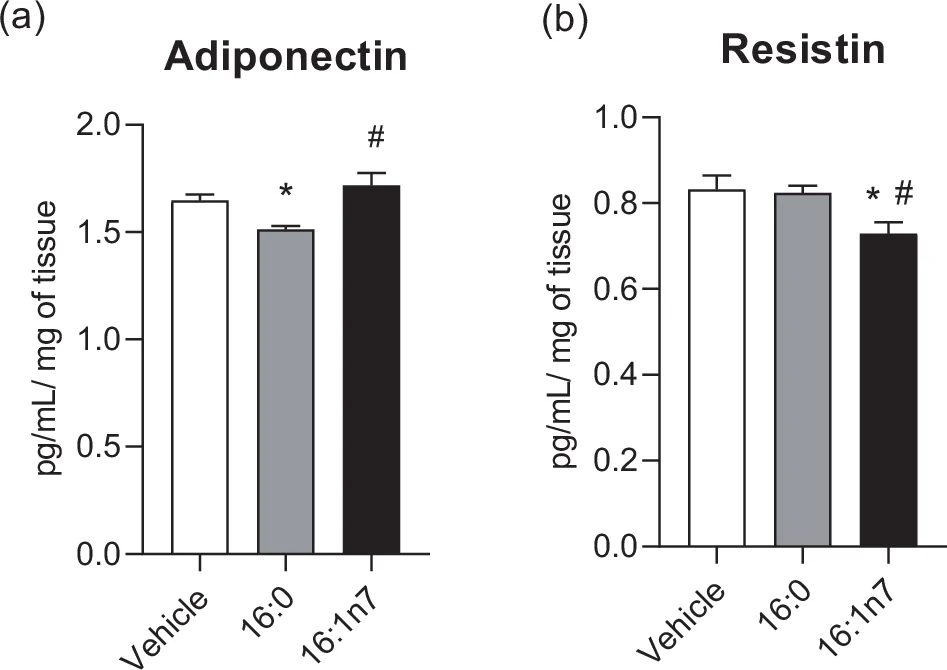
Adiponectin (**a**) and resistin (**b**) secretion into the culture medium from human visceral adipose tissue (vWAT) explants treated for 48 h with either vehicle, palmitic acid (16:0, 200 μM) or palmitoleic acid (16:1n7, 200 μM). Results are means  ±  SEM (*n*  =  5/group). *p  <  0.05 vs. vehicle and ^#^*p*  <  0.05 vs^.^ 16:0

Adipokines involved in the regulation of glucose metabolism were next evaluated (Fig. 3a-b). Adiponectin levels were significantly reduced in vWAT treated with 16:0 (1.50 ± 0.01 pg/mL/mg tissue) compared with vehicle (1.64 ± 0.02 pg/mL/mg tissue) and 16:1n-7 (1.71 ± 0.05 pg/mL/mg tissue), whereas no difference was observed between the 16:1n-7 and vehicle groups. In contrast, resistin levels were significantly lower in the 16:1n-7 treated group (0.72 ± 0.02 pg/mL/mg tissue) than in the vehicle (0.80 ± 0.03 pg/mL/mg tissue) and 16:0 groups (0.82 ± 0.01 pg/mL/mg tissue).

### Palmitoleic acid attenuates oxidative stress in human vWAT

**Fig. 4:**
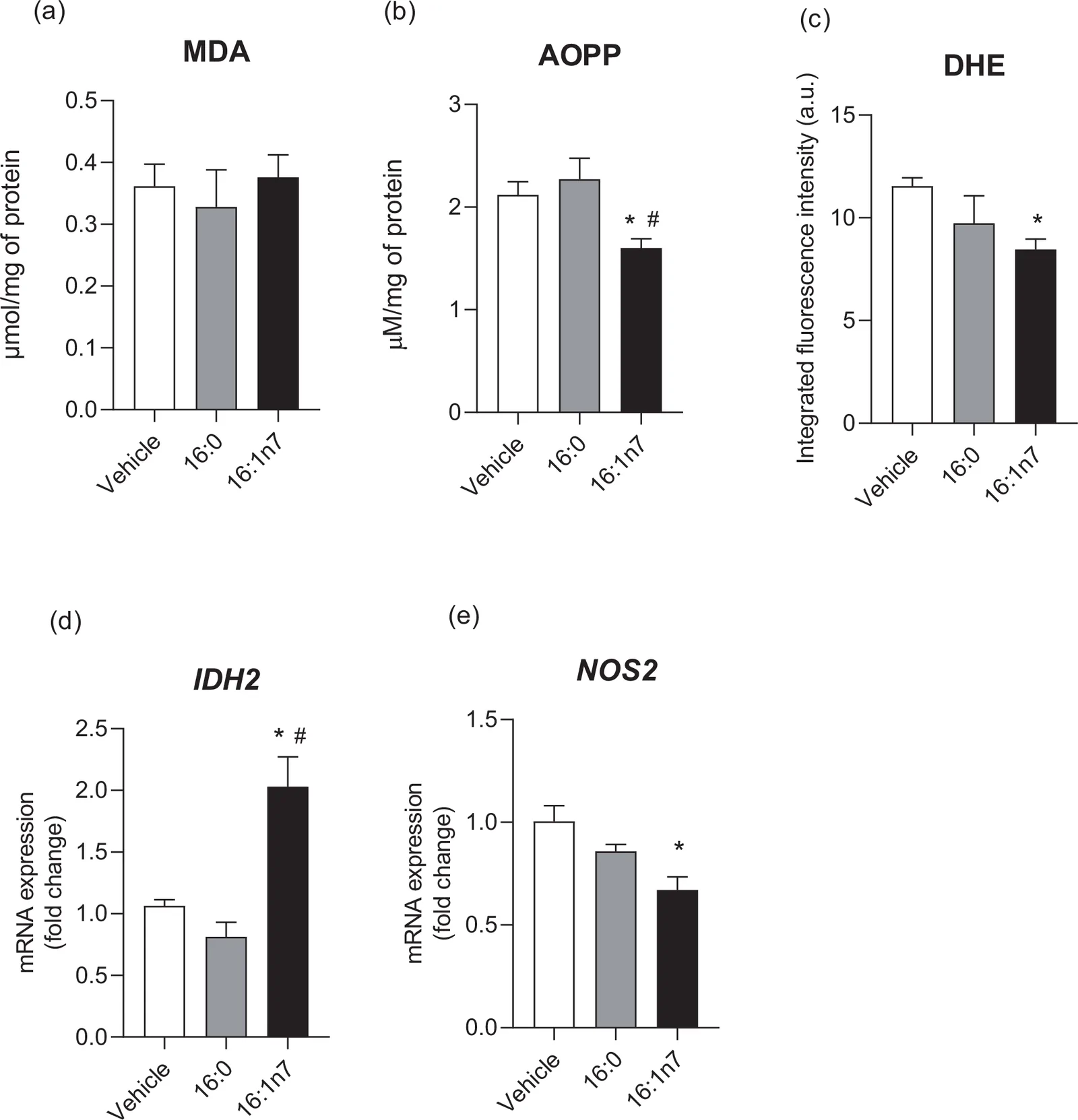
Concentrations of malondialdehyde (MDA, **a**), advanced oxidation protein products (AOPP, **b**), and in situ detection of reactive oxygen species (ROS) by dihydroethidium (DHE) fluorescence (**c**) in human visceral adipose tissue (vWAT) explants; mRNA expression of Isocitrate dehydrogenase 2 (*IDH2*, **d**) and Inducible nitric oxide synthase (*NOS2*, **e**) in isolated human omental adipocytes treated for 48 h with either vehicle, palmitic acid (16:0, 200 μM) or palmitoleic acid (16:1n7, 200 μM). Results are means  ±  SEM (*n*  =  5/group). *p  <  0.05 vs. vehicle and ^#^*p*  <  0.05 vs^.^ 16:0

Given the close relationship between glucose metabolism and redox homeostasis, we next evaluated the effects of 16:1n-7 on oxidative stress related markers in human vWAT. As shown in Fig. 4a, no statistically significant differences were observed in MDA levels among the experimental groups (vehicle: 0.36 ± 0.03; 16:1n-7: 0.37 ± 0.03; 16:0: 0.32 ± 0.06 µmol/mg). However, treatment with 16:1n-7 significantly reduced AOPP levels (1.6 ± 0.08 µM/mg) when compared with both the vehicle group (2.1 ± 0.1 µM/mg) and the 16:0 group (2.2 ± 0.2 µM/mg), Fig. 4b. Moreover, 16:1n-7 treatment markedly decreased ROS production, as assessed by DHE fluorescence (vehicle: 11.5 ± 0.4; 16:0: 9.7 ± 1.3; 16:1n-7: 8.4 ± 0.5 arbitrary units, % area), Fig. 4c.

In parallel, 16:1n-7 significantly increased the expression of *IDH2*, with a 1.91-fold increase relative to the vehicle group and a 2.49-fold increase compared with the 16:0 group (Fig. 4d). Consistently, treatment with 16:1n-7 reduced *NOS2* expression to 0.67-fold of vehicle levels and 0.80-fold of the 16:0 group (Fig. 4e). Together, these findings indicate that 16:1n-7 contributes to the attenuation of oxidative stress in human vWAT.

## Discussion

This study unveils important actions of palmitoleic acid in the regulation of glucose metabolism and oxidative stress in isolated human adipocytes and vWAT explants obtained from women with obesity and prediabetes, in vitro. We found that palmitoleic acid increases basal and insulin-stimulated glucose uptake and enhances the expression of the main glucose transporters, *GLUT1* and *GLUT4*, together with increased AMPKα content. Along with increased uptake, palmitoleic acid enhances the expression of key genes involved in metabolic glucose consumption rather than de novo lipogenesis, modulates adipokine secretion, and contributes to the attenuation of oxidative stress. Altogether, these findings support a role for palmitoleic acid as a metabolic regulator of human adipose tissue under metabolically compromised conditions, thereby extending previous observations from animal models.

Palmitoleic acid is a monounsaturated fatty acid derived endogenously from palmitic acid by stearoyl-CoA desaturase-1 (SCD-1) and obtained exogenously from dietary sources such as macadamia oil, sea buckthorn pulp, coconut oil, and salmon [15, 27]. Experimental studies using in vivo and in vitro models have demonstrated that palmitoleic acid acts as a lipokine with beneficial metabolic actions, including improvements in systemic and local glucose homeostasis [13, 15, 19]. These effects appear to be tissue-specific and mediated by distinct intracellular signaling pathways. In the seminal study by Cao et al. [15], palmitoleic acid improved glucose uptake and insulin sensitivity in skeletal muscle from FABP − /− mice and C2C12 myotubes through enhanced AKT activation, without alterations in glucose transporter expression. In parallel, studies conducted by our group in WAT from C57BL/6 mice and in 3T3-L1 adipocytes demonstrated increased glucose uptake and GLUT4 content, but not GLUT1, through AMPK-dependent pathways, while also redirecting intracellular glucose flux toward energy-producing rather than energy-storing pathways [13, 14].

Here, we demonstrate that palmitoleic acid increases basal and insulin-stimulated glucose uptake, as well as the expression of the glucose transporters GLUT1 and GLUT4, in adipocytes isolated from human vWAT under metabolically compromised conditions.

Adipocyte glucose uptake can occur independently of insulin through constitutively expressed GLUT1, which is localized at the plasma membrane and exhibits relatively low transport capacity for glucose [28–30]. Upon insulin stimulation, however, glucose uptake is markedly amplified due to the translocation of GLUT4 from intracellular storage vesicles to the cell surface. This process is driven by activation of the classical insulin signaling cascade involving the insulin receptor substrate (IRS), phosphoinositide 3-kinase (PI3K), and Akt, resulting in a substantial increase in cellular glucose influx, by 10–20 fold [28–34].

Notably, in this human vWAT model, the metabolic effects of palmitoleic acid occurred in the absence of activation of canonical insulin signaling pathways, as no changes were detected in total AKT or AKT phosphorylation. Instead, palmitoleic acid increased total AMPKα protein content without altering AMPK phosphorylation. These findings are consistent with previous reports indicating that metabolic adaptations in adipocytes may occur independently of AKT activation, particularly in response to lipid-derived signaling molecules [13, 35, 36].

AMPK is a central regulator of glucose metabolism, fatty acid oxidation, and inflammatory responses in adipocytes [37]. In conditions of chronic nutrient excess, such as obesity or prolonged high-fat diet exposure, AMPKα signaling is frequently impaired, a process associated not only with altered phosphorylation dynamics but also with reduced total AMPK protein content, contributing to metabolic dysfunction and insulin resistance. While acute metabolic stress predominantly modulates AMPK activity through phosphorylation, sustained obesogenic environments appear to induce longer-term adaptations at the level of enzyme abundance [36–39].

Fatty acids differentially regulate AMPK signaling. Saturated fatty acids, such as palmitic acid, have been shown to suppress AMPK activity and content, whereas unsaturated fatty acids, including monounsaturated and n-3 polyunsaturated species, may preserve or enhance AMPK expression, supporting cellular energy balance and glucose homeostasis [36, 38, 39]. In this context, the increase in total AMPKα observed in human vWAT treated with palmitoleic acid, in the absence of detectable changes in phosphorylation, suggests a modulation of cellular metabolic capacity as opposed to acute kinase activation. Although protein expression was assessed here by immunohistochemistry, a semiquantitative method, these data nonetheless offer important evidence regarding the differential regulation of AMPKα protein level.

Beyond glucose transport, 16:1n-7 induced a coordinated increase in the expression of adipocyte genes favoring intracellular glucose utilization rather than de novo fatty acid synthesis. The upregulation of *GPD1* and *PCK1* suggests enhanced glyceroneogenic capacity, promoting the conversion of glucose into glycerol-3-phosphate and supporting triglyceride–fatty acid reesterification. Consistently, *GK* expression was preserved in 16:1n-7–treated adipocytes compared with the reduction observed with 16:0, further supporting intracellular glycerol reutilization. Together, these mechanisms may limit excessive fatty acid release and contribute to metabolic homeostasis[9, 40, 41].

In previous experimental studies, we demonstrated that palmitoleic acid increases glycerol-3-phosphate generation concomitantly with increased lipolysis in lean rodents, thereby enhancing the recycling of lipolysis-derived fatty acids into triacylglycerols [13, 14]. In contrast, in the present study using adipocytes from women with obesity and prediabetes, lipolysis, as well as *LIPE* and *PNPLA2* expression, were not altered by 16:1n-7, suggesting a context-dependent metabolic response. Notably, the absence of lipolytic activation in this context may be beneficial, avoiding excessive fatty acid release while supporting a more efficient anabolic handling of glucose within adipocytes.

In addition, 16:1n-7 increased *CS* expression, a gene associated with mitochondrial metabolism. Although mitochondrial function was not directly assessed, this finding may reflect modulation of mitochondrial metabolic pathways, which are often impaired in obesity-associated adipocyte dysfunction [42]. The concomitant upregulation of *PPARG* expression further supports the transcriptional response induced by 16:1n-7. Beyond its well-established role in adipogenesis, PPARγ is also involved in the regulation of glucose metabolism in mature adipocytes, directly influencing insulin sensitivity and glucose uptake through coordinated control of GLUT1 and GLUT4 expression and trafficking. Indeed, suppression of PPARγ has been shown to impair insulin-stimulated glucose uptake by disrupting both GLUT1- and GLUT4-mediated glucose transport in adipocytes [43, 44]. In this context, the parallel increase in *PPARG*, *GLUT1, GLUT4*, and *CS* expression observed in our model suggests coordinated transcriptional changes associated with glucose metabolism and adipocyte metabolic function.

Beyond its effects on intracellular glucose handling and metabolic signaling, 16:1n-7 also modulated the endocrine function of human vWAT. In the present study, 16:1n-7 did not alter adiponectin secretion, whereas 16:0 markedly reduced its levels. In contrast, 16:1n-7 significantly reduced resistin secretion, an adipokine closely linked to insulin resistance, pro-inflammatory signaling and inflammation-related chronic diseases [45, 46]. The reduction of resistin further supports the role of 16:1n-7 in promoting a more favorable metabolic and inflammatory profile, contributing to improved glucose metabolism in adipocytes.

WAT derived from individuals with obesity, particularly under conditions of hyperglycemia and hyperinsulinemia, exhibit increased oxidative damage, mitochondrial dysfunction, pro-inflammatory signaling, and impaired cellular homeostasis compared with WAT from eutrophic individuals [5, 12, 42, 47].These alterations are closely linked to redox imbalance, characterized by a predominance of pro-oxidative processes over antioxidant defenses [5, 11]. Given the tight interconnection between obesity, glucose metabolism, and redox homeostasis, we evaluated the impact of 16:1n-7 on oxidative stress related parameters in human vWAT. In this context, although lipid peroxidation assessed by MDA levels remained unchanged, 16:1n-7 reduced protein oxidation and ROS production, as reflected by lower AOPP levels and decreased DHE fluorescence. These findings indicate that 16:1n-7 selectively mitigates oxidative stress related alterations without uniformly affecting all oxidative markers, underscoring the complexity of redox regulation in vWAT under metabolic stress.

In parallel, 16:1n-7 increased *IDH2* expression, an enzyme involved in NADPH supply and cellular redox control. Although enzymatic activity and mitochondrial function were not directly assessed, increased *IDH2* expression is consistent with a potential reinforcement of cellular redox buffering capacity. IDH2 has been implicated in metabolic regulation under conditions of obesity and insulin resistance [48], and its regulation has been linked to enhanced antioxidant defense mechanisms [49]. Moreover, 16:1n-7 reduced the expression of *NOS2*, an enzyme responsible for NO production from L-arginine that is upregulated under inflammatory conditions and contributes to disturbances in glucose and lipid metabolism [50]. These results indicate that 16:1n-7 reduces oxidative stress in human vWAT by influencing redox balance and inflammatory pathways.

From a translational perspective, this study supports the concept that palmitoleic acid represents a promising functional lipokine capable of exerting metabolically favorable effects on human adipose tissue and isolated adipocytes under conditions of obesity and prediabetes. Notably, 16:1n-7 improved glucose uptake and glucose metabolism while attenuating oxidative stress and promoting a less pro-inflammatory adipokine profile. Importantly, as the adipose samples were derived from women aged 25–40 years, these findings provide preliminary evidence supporting the biological relevance of this naturally occurring lipid within this specific demographic group, particularly considering the higher global prevalence of obesity among women compared with men [51, 52]. Although the in vitro design does not fully capture the complexity of adipose tissue in vivo and mitochondrial function was not directly assessed, future studies should determine whether these cellular effects translate into systemic metabolic benefits.

## Conclusions

In conclusion, palmitoleic acid enhances glucose uptake and metabolism in human visceral adipocytes, favoring intracellular glucose utilization and glyceroneogenesis rather than de novo lipogenesis, without changes in lipolytic activity, and reduces oxidative stress markers in vWAT from women with obesity and prediabetes. These findings identify 16:1n-7 as a bioactive lipid that modulates human adipose tissue function and support further investigation of its therapeutic potential in metabolic disease.

## Supplementary information

Supplementary Information

Supplementary Caption

## Data Availability

The data generated and/or analyzed during this study are included in this published article and are available from the corresponding author upon reasonable request.
