# Supplementary material for: Palmitoleic acid n7 improves glucose uptake and metabolism and attenuates oxidative stress in human visceral adipocytes
Source: Endocrine. 2026 Sep 28;91(1):304. doi: 10.1007/s12020-026-04766-6 (PMC13619680; doi:10.1007/s12020-026-04766-6)
Supplement: Supplementary file 1 [file 12020_2026_4766_MOESM1_ESM.pdf]

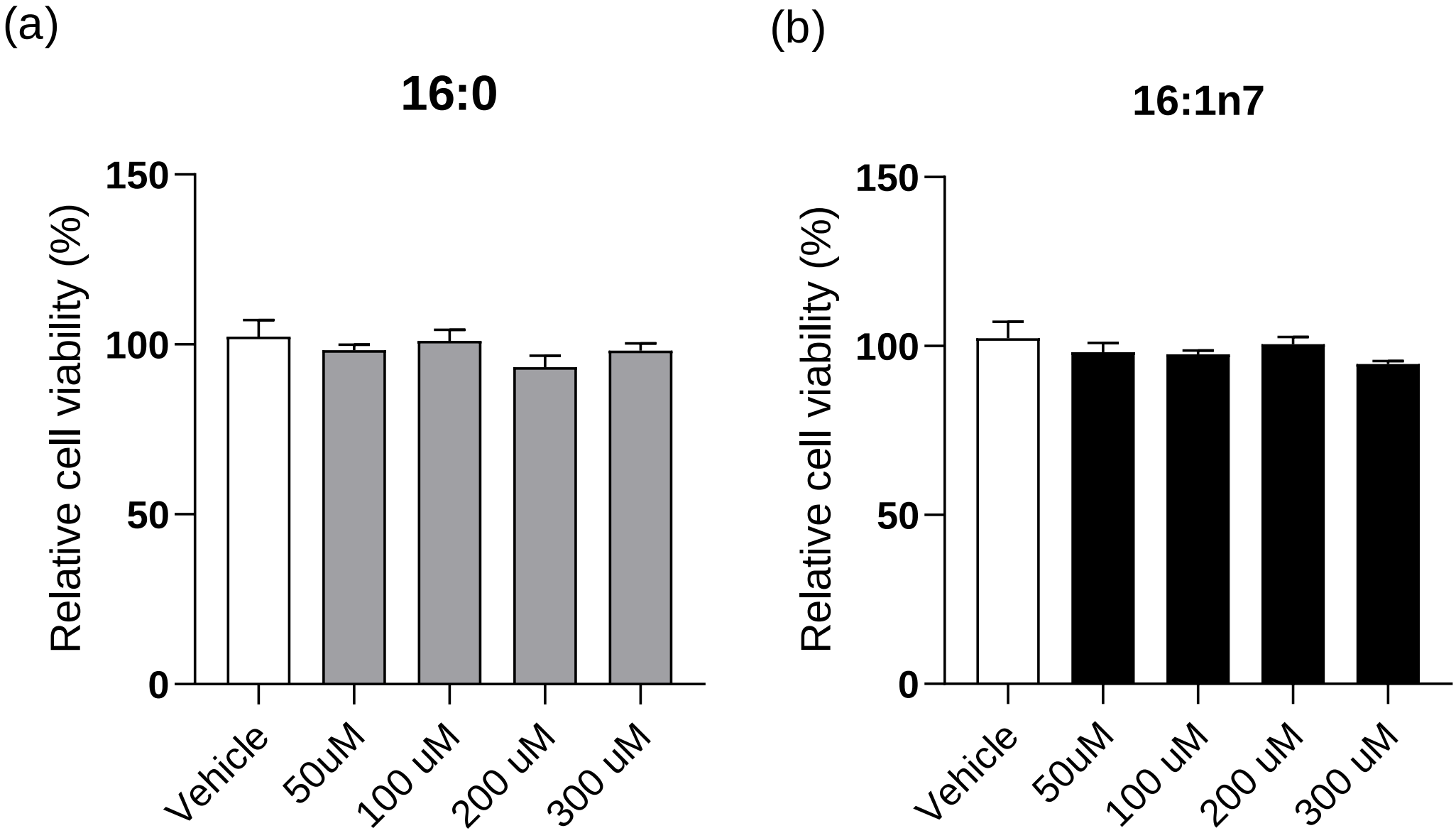
(a)
16:0
Relative cell viability (%)
150
100
50
0
Vehicle
50uM
100 uM
200 uM
300 uM
(b)
16:1n7
Relative cell viability (%)
150
100
50
0
Vehicle
50uM
100 uM
200 uM
300 uM
