# Supplementary material for: Palmitoleic acid n7 improves glucose uptake and metabolism and attenuates oxidative stress in human visceral adipocytes
Source: Endocrine. 2026 Sep 28;91(1):304. doi: 10.1007/s12020-026-04766-6 (PMC13619680; doi:10.1007/s12020-026-04766-6)
Supplement: Supplementary file 2 [file 12020_2026_4766_MOESM2_ESM.docx]

**Supplementary Figure S1**. Cell viability of human visceral adipocytes following treatment with palmitic acid (16:0) or palmitoleic acid (16:1n-7). Adipocytes were treated for 48 h with vehicle or increasing concentrations (50, 100, 200, and 300 μM) of palmitic acid (a) or palmitoleic acid (b). Cell viability was assessed by the MTT assay and expressed as a percentage of the vehicle group. Data are presented as mean ± SEM (n = 5). No significant differences were observed compared with the vehicle group.
